# Supplementary material for: Environmental influences and individual characteristics that affect learner-centered teaching practices
Source: PLoS One. 2021 Apr 30;16(4):e0250760. doi: 10.1371/journal.pone.0250760 (PMC8087079; doi:10.1371/journal.pone.0250760)
Supplement: S1 File — (DOCX) [file pone.0250760.s001.docx]

**S1 File. Relevant Background Survey Questions**

*Please indicate the degree to which you agree or disagree with the following statements*

*(0=strongly disagree, 10=strongly agree).*

- My department is committed to transforming curricula and courses to enhance active learning and inquiry-based teaching.
- I frequently discuss issues pertaining to the improvement of teaching and learning with colleagues in my department.
- Other faculty in my department feel the same as I do about the need to improve undergraduate teaching and learning.
- Faculty in my department collaborate to achieve effective teaching (e.g., design, test, discuss curricula, etc.)
- Faculty in my department are interested in or are already conducting scholarly work about teaching and learning.
- Faculty in my department are recognized, evaluated, and rewarded for effective teaching.

*Please rate your knowledge of, first-hand experience with, and confidence about implementing each of the following.*

Knowledge (Low/Med/High), First-hand experience (Low/Med/High), Confidence (Low/Med/High)

- Course/curriculum planning
- Applying theories of learning (e.g., constructivism) to teaching practice
- Using technology in instruction
- Active learning
- Cooperative learning
- Using case studies
- Problem-based learning
- Inquiry-based teaching
- Assessment
- STEM education reform

*Imagine that you plan to develop and teach a course that uses active learning. Please use the scale below to indicate the degree to which the following would pose a challenge as you implement this course (1=not a challenge, 5=highly challenging). If an item is not applicable in your teaching context, please indicate that with the checkbox.*

1-5, N/A

- Time to plan, develop, and/or adapt materials
- Time to grade and/or give adequate feedback
- Time to train colleagues and/or TAs
- Cooperation/support of faculty in my department
- Cooperation of TAs and/or other instructors
- Support of campus administration
- Recognition or rewards for effective teaching
- Issues with reappointment/promotion
- Student attitudes toward and motivation around non-traditional teaching methods
- Student feedback through course evaluations
- Classroom infrastructure
- Access to instructional technology (e.g., clickers)
