# Supplementary material for: Environmental influences and individual characteristics that affect learner-centered teaching practices
Source: PLoS One. 2021 Apr 30;16(4):e0250760. doi: 10.1371/journal.pone.0250760 (PMC8087079; doi:10.1371/journal.pone.0250760)
Supplement: S2 File — (DOCX) [file pone.0250760.s002.docx]

**S2 File. SCII Data**

| **Column Header** | **Description** |
| --- | --- |
| IntID | Institution ID |
| DeptID | Department ID |
| N Responses | Number of SCII survey responses |
| N Faculty | Number of faculty in the department |
| Percent Response | Percent survey response for the department |
| N Participants | Number of study participants per department |
| Notes | Notes describing N_participants column |

| **IntID** | **DeptID** | **N Responses** | **N Faculty** | **Percent Response** | **N Participants** | **Notes** |
| --- | --- | --- | --- | --- | --- | --- |
| 2037 | 3040 | 9 | 35 | 25.71429 | 2 | One pair of participants |
| 2010 | 3011 | 24 | 52 | 46.15385 | 2 | One pair of participants |
| 2011 | 3012 | 4 | 27 | 14.81481 | 2 | One pair of participants |
| 2011 | 3027 | 8 | 29 | 27.58621 | 2 | One pair of participants |
| 2021 | 3023 | 12 | 54 | 22.22222 | 2 | One pair of participants |
| 2002 | 3002 | 17 | 19 | 89.47368 | 4 | Two pairs of participants |
| 2012 | 3014 | 8 | 24 | 33.33333 | 2 | One pair of participants |
| 2033 | 3036 | 3 | 23 | 13.04348 | 2 | One pair of participants |
| 2013 | 3015 | 10 | 19 | 52.63158 | 2 | One pair of participants |
| 2020 | 3022 | 5 | 13 | 38.46154 | 2 | One pair of participants |
| 2035 | 3038 | 19 | 44 | 43.18182 | 3 | One pair of participants with original comparison faculty dropping out |
| 2001 | 3001 | 6 | 13 | 46.15385 | 2 | One pair of participants |
| 2022 | 3024 | 10 | 9 | 111.1111 | 2 | One pair of participants |
| 2031 | 3033 | 8 | 24 | 33.33333 | 1 | One half of pair |
| 2031 | 3034 | 7 | 16 | 43.75 | 1 | One half of pair |
| 2015 | 3017 | 8 | 29 | 27.58621 | 2 | One pair of participants |
| 2026 | 3028 | 5 | 25 | 20 | 2 | One pair of participants |
| 2029 | 3031 | 5 | 21 | 23.80952 | 2 | One pair of participants |
| 2028 | 3030 | 6 | 23 | 26.08696 | 2 | One pair of participants |
| 2034 | 3037 | 9 | 13 | 69.23077 | 2 | One pair of participants |
| 2030 | 3032 | 15 | 22 | 68.18182 | 4 | Two pairs of participants |
| 2016 | 3018 | 27 | 39 | 69.23077 | 2 | One pair of participants |
| 2039 | 3042 | 11 | 26 | 42.30769 | 2 | One pair of participants |
| 2006 | 3007 | 7 | 44 | 15.90909 | 1 | One half of pair |
| 2006 | 3006 | 10 | 47 | 21.2766 | 1 | One half of pair |
| 2009 | 3010 | 18 | 63 | 28.57143 | 4 | Two pairs of participants |
| 2018 | 3020 | 21 | 42 | 50 | 2 | One pair of participants |
| 2005 | 3005 | 16 | 34 | 47.05882 | 2 | One pair of participants |
| 2008 | 3009 | 16 | 30 | 53.33333 | 2 | One pair of participants |
| 2003 | 3003 | 12 | 21 | 57.14286 | 4 | Two pairs of participants |
| 2007 | 3008 | 12 | 136 | 8.823529 | 4 | Two pairs of participants |
| 2019 | 3021 | 7 | 34 | 20.58824 | 2 | One pair of participants |
| 2038 | 3041 | 7 | 12 | 58.33333 | 2 | One pair of participants |
