# Supplementary material for: Environmental influences and individual characteristics that affect learner-centered teaching practices
Source: PLoS One. 2021 Apr 30;16(4):e0250760. doi: 10.1371/journal.pone.0250760 (PMC8087079; doi:10.1371/journal.pone.0250760)
Supplement: S3 File — (DOCX) [file pone.0250760.s003.docx]

**S3 File. SCII items and exploratory factor analysis loadings for a six factor structure.**

Below are the items that loaded on each factor above 0.3. All loadings are in parentheses following each item.

Factor 1 - **Leadership**

- The department chair…
  - Encourages instructors to go beyond traditional approaches to teaching. (0.684)
  - Has a clear vision of how to improve teaching in the department. (0.826)
  - Implements teaching-related policies in a consistent and transparent manner. (0.767)
  - Inspires respect for his/her ability as a teacher. (0.807)
  - Is receptive to ideas about how to improve teaching in the department. (0.830)
  - Is tolerant of fluctuations in student evaluations when instructors are trying to improve their teaching. (0.588)
  - Is willing to seek creative solutions to budgetary constraints in order to maintain adequate support for teaching improvements. (0.757)

Factor 2 - **Mentoring and Material Support**

- Instructors in my department ARE…
  - Satisfied with their teaching workload. (0.444)
  - Assigned a mentor for advice about teaching. (0.322)
- Instructors in my department HAVE…
  - Adequate departmental funding to support teaching improvement. (0.656)
  - Adequate space to meet with students outside of class. (0.598)
  - Adequate time to reflect upon and make changes to their instruction. (0.739)
  - The support they need to employ educational technologies in their classrooms. (0.538)
- In my department…New instructors are provided with teaching development opportunities and resources. (0.386)

Factor 3 - **Flexibility in Teaching**

- Instructors in my department HAVE…
  - Considerable flexibility in the content they teach in their courses. (0.846)
  - Considerable flexibility in the way they teach their courses. (0.813)

Factor 4 - **Collegiality**

- Instructors in my department…
  - Frequently talk with one another. (0.909)
  - Discuss the challenges they face in the class-room with colleagues. (0.901)
  - Share resources (ideas, materials, sources, technology, etc.) about how to improve teaching with colleagues. (0.705)
  - Aspire to become better teachers. (0.403)

Factor 5 - **Evaluation of Effective Teaching**

- In my department…
  - Applicants for all teaching positions are required to provide evidence of effective teaching. (0.724)
  - Evidence of effective teaching is valued when making decisions about continued employment and/or promotion. (0.875)
  - Teaching effectiveness is evaluated fairly. (0.604)
  - Teaching is respected as an important aspect of academic work. (0.524)
  - All of the instructors are sufficiently competent to teach effectively. (0.482)

Factor 6 - **Support for Teaching Improvement**

- Instructors in my department…
  - Value teaching development services available on campus as a way to improve their teaching. (0.441)
- Instructors in my department ARE…
  - "Ahead of the curve" when it comes to implementing innovative teaching strategies. (0.430)
  - Willing to align the content of their courses to improve student learning. (0.361)
- In my department…There are structured groups organized around the support and pursuit of teaching improvement. (0.516)
