# Supplementary material for: Environmental influences and individual characteristics that affect learner-centered teaching practices
Source: PLoS One. 2021 Apr 30;16(4):e0250760. doi: 10.1371/journal.pone.0250760 (PMC8087079; doi:10.1371/journal.pone.0250760)
Supplement: S4 File — (DOCX) [file pone.0250760.s004.docx]

**S4 File. Correlations between the TBI and ATI instruments**

The Conceptual Change/Student-Focused subscale from the Approaches to Teaching Inventory instrument was significantly correlated with the “Beliefs: Learning Facilitation” (Linear regression, *P* value < 0.001, r^2^ = 0.12) and “Intent: Learning Facilitation” (Linear regression, *P* value < 0.001, r^2^ = 0.41) subscales of the Teaching Beliefs and Intentions instrument. Likewise, the Information Transfer/Teacher-Focused subscales from the ATI was significantly correlated with the “Beliefs: Knowledge Transmission” (Linear regression, *P* value < 0.001, r^2^ = 0.17) and “Intents: Knowledge Transmission” (Linear regression, *P* value < 0.001, r^2^ = 0.24) subscales from the TBI.
