# Supplementary material for: Environmental influences and individual characteristics that affect learner-centered teaching practices
Source: PLoS One. 2021 Apr 30;16(4):e0250760. doi: 10.1371/journal.pone.0250760 (PMC8087079; doi:10.1371/journal.pone.0250760)
Supplement: S5 File — (DOCX) [file pone.0250760.s005.docx]

**S5 File. Data Column Headers**

| **Column Header** | **Description** |
| --- | --- |
| IntID | Institution ID |
| PartID | Participant ID |
| PairID | Pair (FIRST IV & comparison) ID |
| FirstID | FIRST IV or comparison faculty |
| Course_YearID | Year of Study |
| Course_UID | Course unique ID |
| SCII_Factor1 | SCII Factor 1 - Leadership factor (derived from EFA) |
| SCII_Factor2 | SCII Factor 2 - Mentoring and Material Support |
| SCII_Factor3 | SCII Factor 3 - Flexibility in Teaching |
| SCII_Factor4 | SCII Factor 4 - Collegiality |
| SCII_Factor5 | SCII Factor 5 - Evaluation of Effective Teaching |
| SCII_Factor6 | SCII Factor 6 - Support for Teaching Improvement |
| Course_Size | Course enrollment |
| Percent_Research | Percent time allocated to research |
| Percent_Teaching | Percent time allocated to teaching |
| Agree_DeptTransform | Response to: Please indicate the degree to which you agree or disagree with the following statements (S1) |
| Agree_DiscussImprovement | Response to: Please indicate the degree to which you agree or disagree with the following statements (S1) |
| Agree_OtherFac_feel | Response to: Please indicate the degree to which you agree or disagree with the following statements (S1) |
| Agree_FacCollab | Response to: Please indicate the degree to which you agree or disagree with the following statements (S1) |
| Agree_FacInterest | Response to: Please indicate the degree to which you agree or disagree with the following statements (S1) |
| Agree_FacRewarded | Response to: Please indicate the degree to which you agree or disagree with the following statements (S1) |
| Knowledge_ActiveLearn | Knowledge level with respect to Active Learning |
| Knowledge_CoopLearn | Knowledge level with respect to Cooperative Learning |
| Knowledge_Assessment | Knowledge level with respect to Assessment |
| Experience_ActiveLearn | Experience level with respect to Active Learning |
| Experience_CoopLearn | Experience level with respect to Cooperative Learning |
| Experience_Assessment | Experience level with respect to Assessment |
| ALChallenge_TimePlan | Challenges to implementing active learning: Time to plan |
| ALChallenge_TimeGrade | Challenges to implementing active learning: Time to grade |
| ALChallenge_TimeTrain | Challenges to implementing active learning: Time to train colleagues/TAs |
| ALChallenge_FacCoop | Challenges to implementing active learning: Faculty cooperation |
| ALChallenge_TACoop | Challenges to implementing active learning: TA cooperation |
| ALChallenge_CampusSupport | Challenges to implementing active learning: Campus support |
| ALChallenge_TeachRewards | Challenges to implementing active learning: Teaching rewards |
| ALChallenge_Promotion | Challenges to implementing active learning: Promotion |
| ALChallenge_StudentAttitudes | Challenges to implementing active learning: Student attitudes |
| ALChallenge_StudentFeedback | Challenges to implementing active learning: Student feedback |
| ALChallenge_Room | Challenges to implementing active learning: Classroom infrastructure |
| ALChallenge_AccessTech | Challenges to implementing active learning: Access to technology |
| SE_CoursePlanning | Self-Efficacy subscale: Course planning |
| SE_TeachMethods | Self-Efficacy subscale: Teaching methods |
| SE_CreateLearnEnv | Self-Efficacy subscale: Creating a learning environment |
| SE_AssessStudentLearn | Self-Efficacy subscale: Assessing student learning |
| SE_InteractwStudents | Self-Efficacy subscale: Interacting with students |
| SE_MasterSubjKnow | Self-Efficacy subscale: Mastery of subject knowledge |
| TBI_B_LF | Teaching Beliefs & Intentions: Belief: Learning Facilitation |
| TBI_B_KT | Teaching Beliefs & Intentions: Belief: Knowledge Transmission |
| TBI_I_LF | Teaching Beliefs & Intentions: Intentions: Learning Facilitation |
| TBI_I_KT | Teaching Beliefs & Intentions: Intentions: Knowledge Transmission |
| RTOP_Score | Mean Reformed Teaching Observation Protocol score |
