# Supplementary material for: Environmental influences and individual characteristics that affect learner-centered teaching practices
Source: PLoS One. 2021 Apr 30;16(4):e0250760. doi: 10.1371/journal.pone.0250760 (PMC8087079; doi:10.1371/journal.pone.0250760)

**S1 Fig. Knowledge about teaching strategies.** These are faculty responding to their knowledge level with respect to active learning, assessment, and cooperative learning. The percentage is the percent of faculty reporting a certain level of knowledge within a group (FIRST IV or comparison).


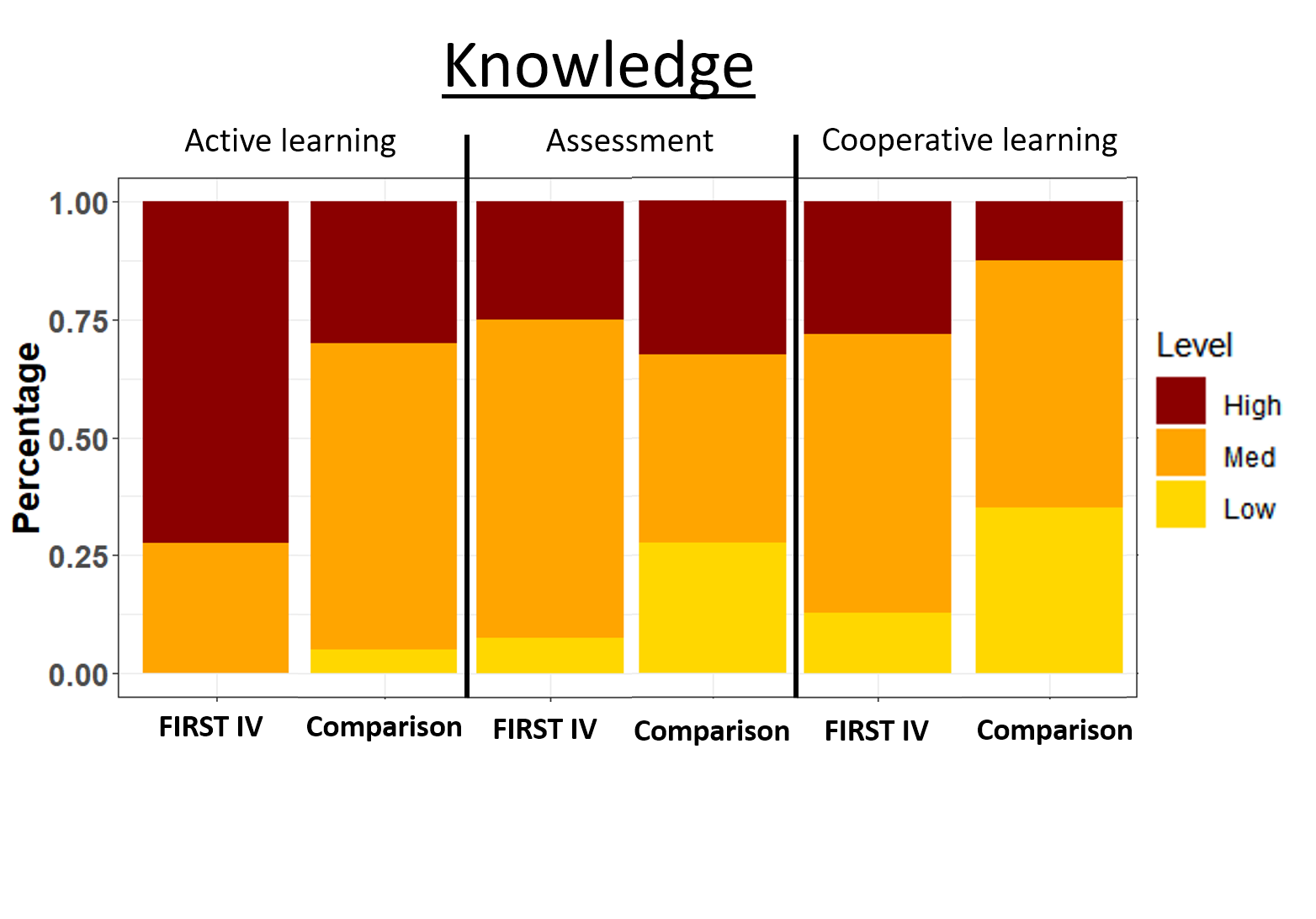

Supplement: S1 Fig — These are faculty responding to their knowledge level with respect to active learning, assessment, and cooperative learning. The percentage is the percent of faculty reporting a certain level of knowledge within a group (FIRST IV or comparison). (DOCX) [file pone.0250760.s006.docx]
