# Supplementary material for: Environmental influences and individual characteristics that affect learner-centered teaching practices
Source: PLoS One. 2021 Apr 30;16(4):e0250760. doi: 10.1371/journal.pone.0250760 (PMC8087079; doi:10.1371/journal.pone.0250760)

**S2 Fig. Experience about teaching strategies.** These are faculty responding to their experience level with respect to active learning, assessment, and cooperative learning. The percentage is the percent of faculty reporting a certain level of experience within a group (FIRST IV or comparison).


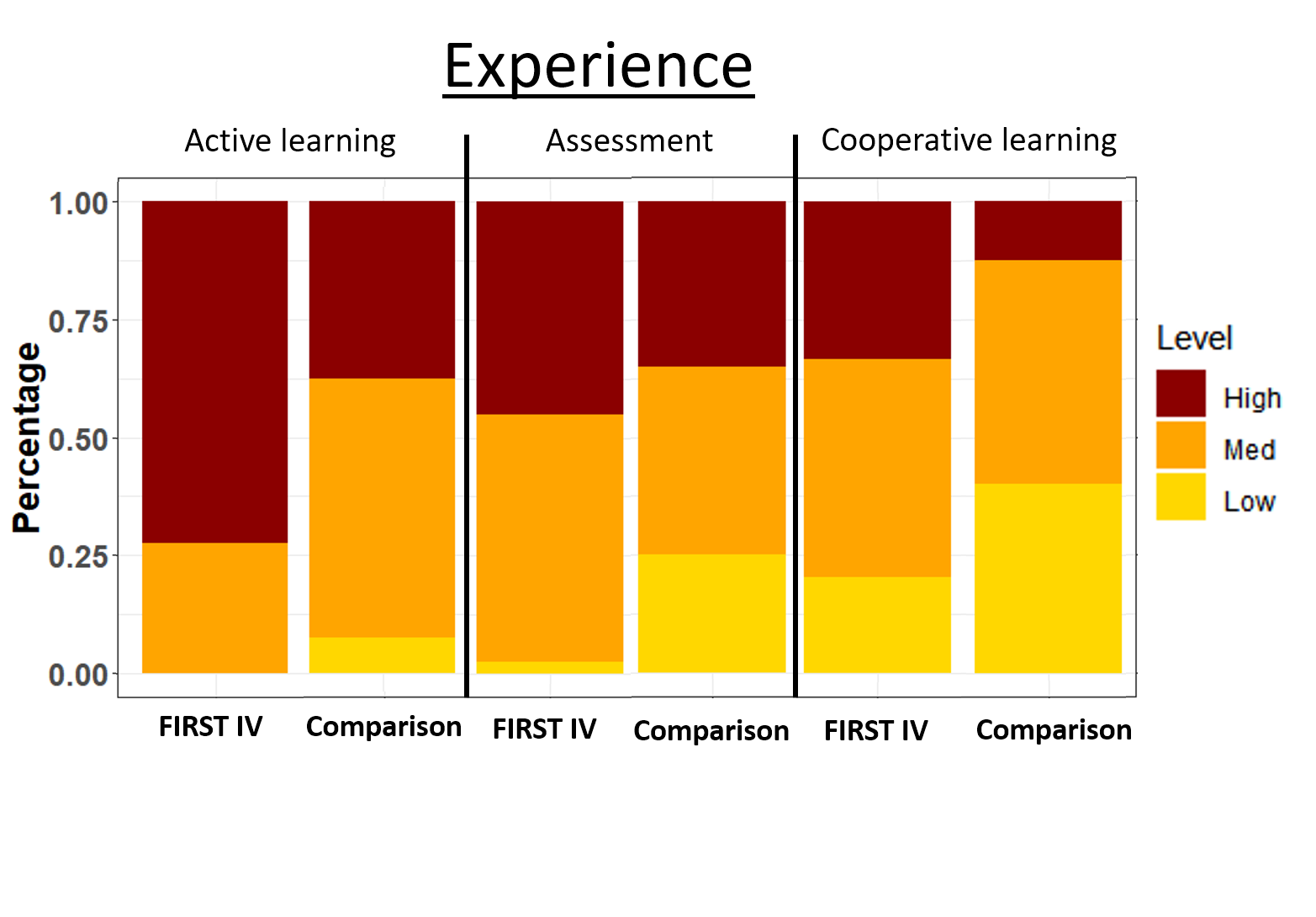

Supplement: S2 Fig — These are faculty responding to their experience level with respect to active learning, assessment, and cooperative learning. The percentage is the percent of faculty reporting a certain level of experience within a group (FIRST IV or comparison). (DOCX) [file pone.0250760.s007.docx]
