# Supplementary material for: Environmental influences and individual characteristics that affect learner-centered teaching practices
Source: PLoS One. 2021 Apr 30;16(4):e0250760. doi: 10.1371/journal.pone.0250760 (PMC8087079; doi:10.1371/journal.pone.0250760)
Supplement: S1 Table — AICc tables for DeltaRTOP & RTOP overall models. (DOCX) [file pone.0250760.s008.docx]

**S1 Table. Model outputs.** AICc tables for DeltaRTOP & RTOP overall models

For Table 4

| **Change in RTOP Model Selection Table** | |  |  |  |  |  |  |  |  |
| --- | --- | --- | --- | --- | --- | --- | --- | --- | --- |
| **Intercept** | **Course Size** | **Percent Teaching** | **Self-Efficacy: Teaching Methods** | **Intentions: Knowledge Transmission** | **df** | **log-likelihood** | **AICc** | **delta** | **weight** |
| -0.014 |  |  | 0.332 |  | 3 | -41.885 | 90.7 | 0 | 0.285 |
| 0.13 |  |  |  |  | 2 | -43.514 | 91.5 | 0.8 | 0.192 |
| -0.085 |  |  | 0.337 | -0.243 | 4 | -41.011 | 91.6 | 0.9 | 0.182 |
| 0.063 |  |  |  | -0.237 | 3 | -42.77 | 92.4 | 1.77 | 0.116 |
| -0.027 | -0.126 |  | 0.362 |  | 4 | -41.479 | 92.5 | 1.84 | 0.114 |
| -0.033 |  | 0.162 | 0.332 |  | 4 | -41.513 | 92.6 | 1.9 | 0.11 |

For Table 5

| **Overall RTOP Model Selection Table** | |  |  |  |  |  |  |  |  |  |
| --- | --- | --- | --- | --- | --- | --- | --- | --- | --- | --- |
| **Intercept** | **Self-Efficacy:**  **Teaching Methods** | **Intentions:**  **Knowledge Transmission** | **Percent Teaching** | **FIRST IV / Comparison** | **SCII: Mentoring and Material Support** | **df** | **log-likelihood** | **AICc** | **delta** | **weight** |
| 0.2646 | 0.434 | -0.344 | -0.212 | + | 0.158 | 7 | -63.8 | 143.6 | 0 | 0.62 |
| 0.2561 | 0.454 | -0.361 | -0.189 | + |  | 6 | -65.53 | 144.5 | 0.98 | 0.38 |
